# Trehalose Inhibits Inflammatory Responses through Mitochondrial Reprogramming in RAW 264.7 Macrophages

**DOI:** 10.3390/antiox12061166

**Published:** 2023-05-28

**Authors:** Seungmin Yu, Hyejeong Park, Wooki Kim

**Affiliations:** 1 Personalized Diet Research Group, Korea Food Research Institute (KFRI), Wanju 55365, Republic of Korea; Y.seungmin@kfri.re.kr; 2 Department of Food Science and Biotechnology, Graduate School of Kyung Hee University, Yongin 17104, Republic of Korea; 9601ys@khu.ac.kr

**Keywords:** anti-inflammatory, macrophage, metabolic reprogramming, oxidative phosphorylation, glycolysis

## Abstract

Studies reported the beneficial effects of trehalose on metabolic syndromes, hyperlipidemia, and autophagy, but its action mechanisms are still poorly understood. Even though trehalose is digested by disaccharidase and absorbed in the intestine, intact molecules encounter immune cells which form a solid balance between the allowance of nutritive substances and the removal of harmful pathogens. In this regard, the polarization of intestinal macrophages into an anti-inflammatory phenotype through metabolic regulation is emerging as a therapeutic strategy for the prevention of gastrointestinal inflammation. The current study investigated the effects of trehalose on immunological phenotypes, energy metabolism, and LPS-induced macrophage mitochondrial functioning. Results indicate that trehalose reduces prostaglandin E_2_ and nitric oxide, which are inflammatory mediators of LPS-induced macrophages. In addition, trehalose further significantly suppressed inflammatory cytokines and mediators via energy metabolism reprogramming towards M2-like status in LPS-stimulated macrophages.

## 1. Introduction

The gastrointestinal tract is open to foodborne microbes and viruses, and a tremendous number of immune cells dwell in it and are finely regulated. The intestinal immune system requires a delicate immune balance between the removal of pathogenic antigens and the allowance of symbiotic microbes [1]. In this regard, intestinal macrophages, which have plasticity in polarization to either pro- or anti-inflammatory subsets, play an important role in maintaining tissue homeostasis by mediating inflammation and resolution [2]. In healthy conditions, blood-circulating monocytes infiltrate the lamina propria and further differentiate into alternatively activated M2-like CD64^+^CD206^hi^CD209^hi^ macrophages [3]. However, dysregulation of homeostasis in digestive inflammation, as exemplified by ulcerative colitis and Crohn’s disease, tips the differentiation of macrophages towards the classically activated M1-like CD11c^hi^CX_3_-chemokine receptor 1-positive (CX_3_CR1^+^) cell phenotype by tumor necrosis factor (TNF) and interleukin (IL)-23 [3,4]. Thus, the activation of macrophages into anti-inflammatory M2 cells in the digestive tract is an emerging dietary strategy for the prevention of intestinal inflammation.

Previous studies have demonstrated that the metabolic pathways of cellular energy production are intrinsically relevant to the functioning of macrophages [5,6]. Specifically, inflammatory M1 macrophages depend on enhanced glycolysis and further lactate fermentation in the cytosol, with blunting mitochondrial oxidative phosphorylation (OXPHOS) through the induction of hypoxia-inducible factor (HIF)-1α [7]. Anti-inflammatory M2 macrophages, however, reciprocally utilize oxidative phosphorylation via fatty acid oxidation (FAO) for continuous energy acquisition [8]. The phenotype switching of macrophages between M1 vs. M2 cells is controlled by mitochondrial function, which can be reprogrammed by dietary approaches.

In the early 20st century, microbial and enzymatic production of trehalose, α-D-glucopyranosyl-(1→1)-α-D-glucopyranoside, was introduced for a replacement of sucrose (β-D-fructofuranosyl-(2→1)-α-D-glucopyranoside). Studies revealed that trehalose has health-promoting properties including anti-inflammatory [9], sperm-protecting [10], and neuro-protecting effects [11]. Furthermore, the remodeling of human microbiota by trehalose was reported [12], indicating that some of the dietary sugars affect the components of digestive tracts including macrophages. Therefore, the current study investigated the anti-inflammatory role of trehalose in murine macrophages with a focus on immunophenotype, energy metabolism, and mitochondrial function. An isomeric disaccharide sucrose and a sugar alcohol xylitol served as controls to highlight the trehalose-specific effects.

## 2. Materials and Methods

### 2.1. Materials

Sucrose, xylitol, and trehalose were purchased from Sigma-Aldrich (St. Louis, MO, USA) and dissolved in distilled water (DW) to prepare a stock solution at a concentration of 1 M. Each stock solution was further diluted in a complete cell culture medium to a final concentration of 1 mM and treated with RAW 264.7 cells.

The RAW 264.7 cell line, originating from murine macrophages, was obtained from the Korean Collection for Type Cultures (KCTC, Jeongeup, Republic of Korea). The cells were maintained in Dulbecco’s modified Eagle’s medium (DMEM) containing 10% fetal bovine serum (FBS) plus 1% antibiotic/antimycotic solution (100 U/mL penicillin, 100 μg/mL streptomycin sulfate, and 0.25 μg/mL amphotericin B). All requisites for cell culture were purchased from Welgene (Gyeongsan, Republic of Korea). Cells were seeded on a 24-well cell culture plate and incubated in 37 °C humidified air at 5% CO_2_ for 24 h following treatment with 1 mM of trehalose, xylitol, or sucrose. Then, the cells were stimulated with 500 ng/mL of lipopolysaccharide (LPS) derived from *Escherichia coli* O55:B5 (Sigma-Aldrich) and further incubated for 24 h to induce acute inflammatory responses.

### 2.2. Quantitative Reverse Transcription Polymerase Chain Reaction (qRT-PCR) Assay

Following LPS stimulation, cells were collected by centrifugation at 300× *g* for 5 min, and total RNA was isolated from cells using an MG Total RNA Extraction Kit (MG Med, Seoul, Republic of Korea) according to the manufacturer’s instructions. The RNA purity and concentration were measured using a NanoDropTM One Microvolume UV-Vis Spectrophotometer (Thermo Fisher Scientific, Waltham, MA, USA). The transcription of pro-inflammatory genes, interleukin-6 (*Il-6*), tumor necrosis factor-α (*Tnf-α*), cyclooxygenase-2 (*Cox-2*), and inducible nitric oxide synthase (*inos*) was assessed by qRT-PCR using an MG One-Step RT-PCR MasterMix (SYBR Green, MG Med) and CFX ConnectTM Real-Time PCR detection system (Bio-Rad, Hercules, CA, USA). The following thermal cycling conditions were applied: 50 °C for 30 min, then 95 °C for 10 min, and then 40 cycles of 95 °C for 5 s and 60 °C for 40 s. The primers used for qRT-PCR are listed in Table 1. Eukaryotic translation elongation factor 2 (*Eef2*) was used as an internal reference gene. The relative transcription levels of pro-inflammatory genes were calculated by using the delta-delta Ct method (2−∆∆Ct) as previously described [13].

### 2.3. Pro-Inflammatory Cytokine and PGE_2_ Quantification

Cell culture supernatants were assessed for pro-inflammatory cytokines (IL-6 and TNF-α) and PGE_2_ by enzyme-linked immunosorbent assay (ELISA). Commercially available sandwich ELISA kits (BD Bioscience, San Jose, CA, USA) were used to quantify pro-inflammatory cytokines according to the manufacturer’s instructions. In brief, the capture antibodies appropriate for each cytokine were coated in a Costar^®^ 96 well EIA/RIA plate (Corning, NY, USA) and incubated overnight at 4 °C. After treatment with the provided assay diluent to block non-specific binding, cell supernatants and each cytokine standard were treated at the designated concentration. Following incubation at room temperature, plates were rinsed with the provided washing buffer, and captured cytokines were further incubated with a mixture of biotinylated detection antibodies and streptavidin-horse radish peroxidase conjugates (sAv-HRP). Then, the substrate solution was treated for the enzymatic reaction, and 1M H_3_PO_4_ was added to stop the reaction, followed by measuring absorbance with a microplate reader (Bio-Rad) at 450 nm.

In PGE_2_ quantification, the competitive ELISA kit (Abcam, Cambridge, UK) was used following the manufacturer’s instructions. Briefly, cell supernatants and standard solution were aliquoted in the provided microplate coated with goat anti-mouse IgG at the designated concentration. Then, alkaline phosphatase (AP) conjugate and PGE_2_ antibody were sequentially added, followed by incubation at room temperature. After a series of washing steps with the provided buffer, *p*-nitrophenylphosphate (pNPP) substrate solution was added, and the provided stop solution was applied. Following the final reaction steps, absorbance was read with a microplate reader (Bio-Rad, Hercules, CA, USA) at 405 nm.

### 2.4. Nitric Oxide (NO) Quantification

The concentration of NO in the cell culture supernatant was quantified using the Promega Griess reagent (Madison, WI, USA) according to the manufacturer’s instructions. Simply, 100 μL of mixed Griess reagent was applied to react with each cell culture supernatant in a 96-well microplate for 10 min at room temperature. The quantification of NO concentration was calculated by comparing the absorbance at 540 nm with a sodium nitrite (NaNO_2_) standard curve.

### 2.5. Assessment of Toll-Like Receptor 4 (TLR4) Endocytosis

Cells were collected following treatment with LPS and divided into two groups to measure surface Toll-like receptor 4 (TLR4) or intracellular TLR4 expression. In surface TLR4 staining, cells were washed twice with pre-chilled PBS and incubated with anti-mouse CD16/32 antibody (Fc block; eBioscience, San Diego, CA, USA) at 4 °C for 15 min to block non-specific binding. After the cells were rinsed with PBS, they were further stained with phycoerythrin (PE)-conjugated anti-mouse TLR4 antibody (TLR4-PE; eBioscience) for 30 min at 4 °C in the dark. Cells were then washed and resuspended with PBS. To evaluate the total amount of TLR4, we applied the cells to the abovementioned surface TLR4 staining, followed by subsequent intracellular TLR4 staining. In brief, surface TLR4-stained cells were fixed and permeabilized using the intracellular fixation and permeabilization buffer set (eBioscience) following the manufacturer’s instructions. Subsequently, cells were stained with TLR4-PE antibody and collected in PBS. The expressions of surface or total TLR4 were determined by mean fluorescence intensity (MFI) using a flow cytometer (Accuri™ C6; BD Bioscience, Mississauga, ON, Canada). TLR4 endocytosis was then calculated using the following equation:

$$\mathrm{TLR}4\;\mathrm{endocytosis}\;\left(\%\right)=\frac{{(\mathrm{MFI}}_{\mathrm{total}\mathrm{TLR}4\;}-\;{\mathrm{MFI}}_{\mathrm{unstained}})\;-{(\mathrm{MFI}}_{\mathrm{surface}\;\mathrm{TLR}4}-\;{\mathrm{MFI}}_{\mathrm{unstained}})}{{\mathrm{MFI}}_{\mathrm{total}\mathrm{TLR}4\;}-\;{\mathrm{MFI}}_{\mathrm{unstained}}}\;\times 100$$

### 2.6. Measurement of Nuclear Factor-Kappa B (NF-κB) Phosphorylation

Following LPS stimulation for 24 h, cell lysates were collected using cell lysis buffer (Cell Signaling Technology, Danvers, MA, USA) following the manufacturer’s instructions. Pathscan^®^ total or phospho-NF-κB (Ser536) sandwich ELISA kits were used to measure total and phospho- NF-κB, respectively. All assay procedures for the NF-κB immunoassay were followed according to the provided instructions from the manufacturer. After the reaction was stopped, sample absorbance was measured at 450 nm using a microplate reader, and the level of NF-κB phosphorylation was calculated as the ratio of the levels of phospho-NF-κB to total NF-κB in each cell lysate.

### 2.7. Metabolic Extracellular Flux Analysis

To assess metabolic phenotypes, such as oxidative phosphorylation (OXPHOS) and glycolysis, the real-time oxygen consumption rate (OCR) and proton efflux rate (PER) were determined using a Seahorse XF extracellular flux analyzer (Agilent Technologies, Palo Alto, CA, USA). The cells were seeded in an XF cell culture plate (Agilent Technologies) and further stimulated with LPS at 500 ng/mL for 24 h. Following stimulation, the plate was incubated for 1 h at 37 °C in a non-CO_2_ incubator to degas. The assay was conducted in a non-buffered XF basal medium (Agilent Technologies) supplemented with 25 mM glucose, 4 mM glutamine, and 1 mM sodium pyruvate according to the manufacturer’s instructions. In response to mitochondrial OXPHOS, OCR was recorded on four consecutive instances in the basal state and after sequential injection of three electron transport chain (ETC) complex inhibitors as follows: 1 μM oligomycin, 1 μM 4-(trifluoromethoxy)phenylhydrazone (FCCP), and 0.5 μM rotenone/antimycin A (Agilent Technologies). Mitochondrial respiratory parameters (basal respiration, maximal respiration, and ATP production) were calculated using the following equations:

Basal respiration = OCR before oligomycin treatment − non-mitochondrial OCR Maximal respiration = OCR after FCCP treatment − non-mitochondrial OCR ATP production = basal OCR − OCR after oligomycin treatment

Regarding the glycolytic rate analysis, the extracellular acidification rate (ECAR) was measured at the basal status and after sequential treatment with 5 μM oligomycin and 500 mM 2-deoxyglucose (2-DG). ECAR was converted into PER to calculate the glycolytic proton efflux rate (glycoPER) as previously described [14]. The characteristics of glycolysis (basal glycolysis and compensatory glycolysis) were calculated using the following formulas:

Basal glycolysis = glycoPER before oligomycin treatment Compensatory glycolysis = Maximum glycoPER after rotenone/antimycin A treatment

After measurement, cells were further assessed for protein content by bicinchoninic acid (BCA) assay, and all data were normalized to protein content (μg protein).

### 2.8. Quantification of Mitochondrial Mass, Potential, and ROS

After LPS stimulation, sucrose, xylose, or trehalose-treated cells were collected and stained with 100 nM MitoTracker™ Green FM (Invitrogen, Carlsbad, CA, USA), 100 nM MitoTracker™ Red CMXRos (Invitrogen), and 5 μM MitoSOX™ Red (Invitrogen) for 15 min at 37 °C according to the manufacturer’s instructions to quantify mitochondrial mass, potential, and ROS, respectively. Then, cells were washed with PBS to remove excess dye and resuspended. The MFI of the stained cells was determined using a flow cytometer (Accuri™ C6; BD Bioscience).

### 2.9. Intracellular ROS Quantification

Intracellular ROS were quantified using 2′,7′-dichlorofluorescin diacetate (DCFDA, Abcam). In brief, RAW 264.7 cells were pre-incubated with trehalose, xylitol or sucrose for 24 h and then treated with LPS for 24 h. Following stimulation, cells were stained with 20 μM DCFDA and incubated for 30 min at 37 °C according to instructions provided by the manufacturer. Then, cells were washed with PBS and resuspended, followed by MFI measurement using a flow cytometer (Accuri™ C6; BD Bioscience).

### 2.10. Statistical Analysis

Data are indicated as mean ± standard error of the mean (SEM) of at least triplicates. Statistical significance was determined by one-way analysis of variance (ANOVA) for multiple comparisons with Tukey’s post hoc test using Prism software (GraphPad Software, San Diego, CA, USA). Significant differences are indicated with different letters, and *p*-values less than 0.05 were considered statistically significant.

## 3. Results

### 3.1. Modulation of Inflammatory Cytokines by Trehalose

**Figure 1:**
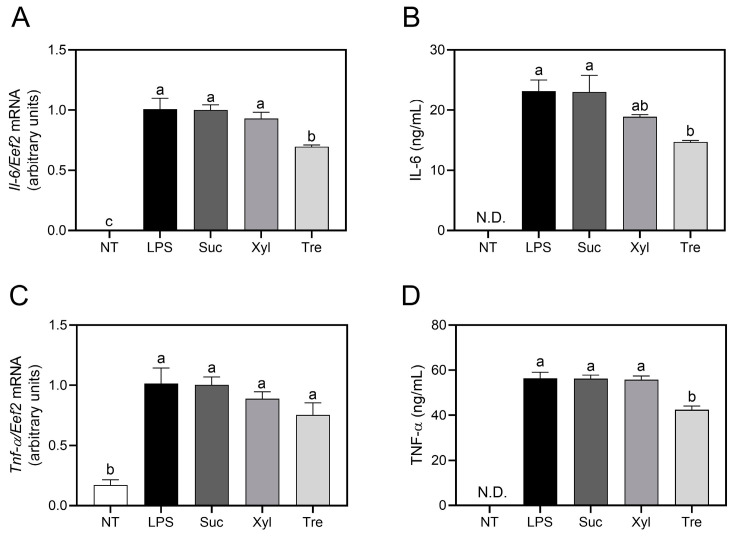
Pro-inflammatory gene transcription and cytokine secretion. The transcription of pro-inflammatory genes ((**A**) *Il-6* and (**C**) *Tnf-α*) and the production of cytokines ((**B**) IL-6 and (**D**) TNF-α) were assessed in RAW 264.7 cells by qRT-PCR and ELISA, respectively. The relative expression of mRNA (**A**,**C**) was determined using the delta-delta Ct method normalizing to the internal *Eef* gene. Data are presented as mean ± SEM (*n* = 4). Different letters indicate significantly different values (*p* < 0.05) as determined by one-way ANOVA followed by Tukey’s posthoc test. NT, non-treated; Suc, sucrose; Xyl, xylitol; Tre, trehalose; N.D., not detected.

To determine the appropriate treatment concentration of the sugar replacements, cell viability was measured by MTT assay at concentrations ranging from 10 μM to 10 mM. Subsequent experiments were carried out using a treatment concentration of 1 mM for the sugar replacements, based on the MTT results described earlier (Figure S1). In inflammatory macrophages stimulated using LPS at 500 ng/mL for 24 h, the transcription of *Il-6* mRNA was significantly down-regulated by trehalose compared to that of cells treated with sucrose or xylitol (Figure 1A). Similar to the mRNA transcription results, trehalose significantly inhibited the production of pro-inflammatory cytokine IL-6 (14.74 ± 0.23 ng/mL) compared to sucrose (23.05 ± 2.77 ng/mL, *p* < 0.05), but xylitol exhibited comparable effect to the LPS-treated control (23.18 ± 1.86 ng/mL) and sucrose (Figure 1B). The transcription of *Tnf-α* was not altered by sucrose or xylitol (Figure 1C), but trehalose-treated cells secreted a significantly reduced amount of TNF-α (42.5 ± 1.65 ng/mL) in the medium compared to sucrose (56.21 ± 1.65 ng/mL, *p* < 0.05) or xylitol (55.76 ± 1.64 ng/mL, *p* < 0.05) (Figure 1D). These results indicate that trehalose, as compared to sucrose and xylitol, specifically reduces pro-inflammatory cytokine production in LPS-induced macrophages.

### 3.2. Suppressive Effects of Trehalose on Inflammatory Mediator Production

**Figure 2:**
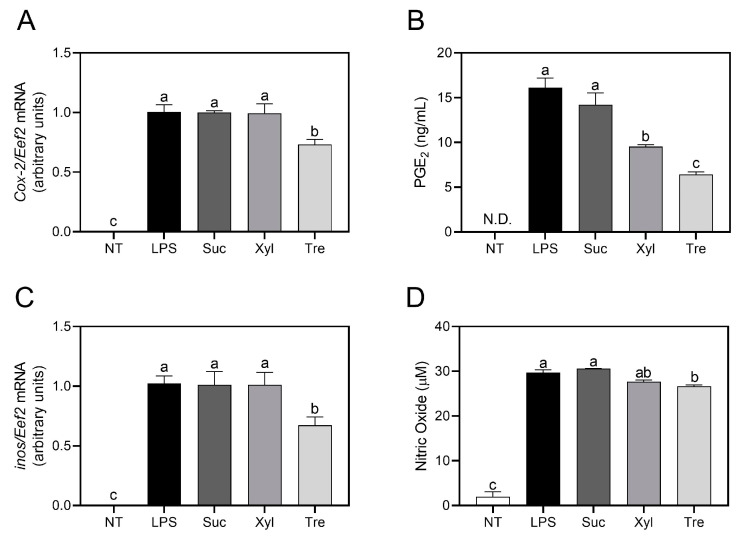
Inflammatory gene transcription and inflammatory mediator production. The transcription of inflammatory genes ((**A**) *Cox-2* and (**C**) *inos*) was assessed in RAW 264.7 cells by qRT-PCR. (**B**) The production of PGE_2_ and (**D**) nitric oxide was quantified by ELISA and colorimetric assay using the Griess reagent, respectively. The relative expression of mRNA (**A**,**C**) was determined using the delta-delta Ct method normalizing to the internal *Eef* gene. Data are presented as mean ± SEM (*n* = 4). Different letters indicate significantly different values (*p* < 0.05) as determined by one-way ANOVA followed by Tukey’s posthoc test. NT, non-treated; Suc, sucrose; Xyl, xylitol; Tre, trehalose; N.D., not detected.

The production of non-protein inflammatory mediators, i.e., eicosanoid PGE_2_ and arginine metabolite nitric oxide (NO), as well as the transcription of their converting enzyme genes (*Cox-2* and *inos*, respectively) was further examined. Compared to the sucrose or LPS-treated control, trehalose significantly inhibited the transcription of *Cox-2* (Figure 2A), yet xylitol exhibited a comparable value to sucrose. The production of PGE_2_ was down-regulated by both trehalose (6.44 ± 0.33 ng/mL) and xylitol (9.54 ± 0.22 ng/mL), where the significance (*p* < 0.05) between the two was also observed (Figure 2B).

Parallel results were obtained regarding the transcription of *inos* (Figure 2C), where trehalose, not xylitol, significantly suppressed transcription compared to LPS-treated control and sucrose. The production of NO revealed a trehalose-specific reduction to 26.64 ± 0.29 μM as compared to that of sucrose (30.60 ± 0.07 μM), but xylitol-treated cells (27.7 ± 0.36 μM NO) exhibited no significance (*p* > 0.05) to both sucrose and trehalose (Figure 2D).

### 3.3. Reduced NF-κB Phosphorylation by Trehalose in LPS-Induced Macrophages

**Figure 3:**
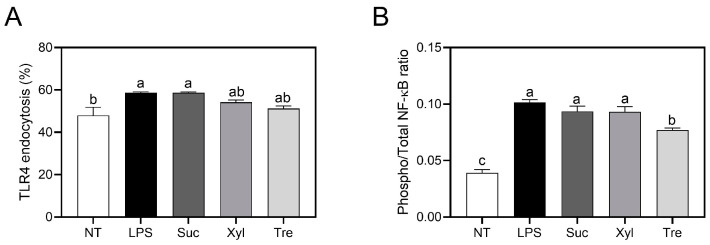
TLR4 endocytosis and NF-κB phosphorylation. (**A**) the endocytosis of TLR4 was assessed by staining with fluorescence-conjugated specific antibody and analyzed by a flow cytometer. (**B**) The phosphorylation of NF-κB was calculated as the ratio of phosho-NF-κB to the total NF-κB as determined by specific ELISA kits and normalization to the total protein. Data are presented as mean ± SEM (*n* = 4). Different letters indicate significantly different values (*p* < 0.05) as determined by one-way ANOVA followed by Tukey’s posthoc test. NT, non-treated; Suc, sucrose; Xyl, xylitol; Tre, trehalose.

Given that the production of pro-inflammatory cytokines and other inflammatory mediators was modulated by trehalose, the upper stream, i.e., TLR4/NF-κB pathway was examined. Macrophages treated with LPS exhibited a significant increase in both TLR4 endocytosis and NF-κB phosphorylation (Figure 3). As shown in Figure 3A, treatment with either xylitol trehalose did not affect TLR4 endocytosis compared to LPS-stimulated control and non-treated cells. In contrast, the phosphorylation of NF-κB, a key cell signaling molecule under TLR4 activation in macrophages, was significantly down-regulated by trehalose as compared to sucrose or xylitol-treated cells (Figure 3B). Trehalose treatment did not affect the total NF-κB expression, as observed in other LPS-treated and untreated cells (Figure S2A). However, it exerted a significant decrease in the expression of phospho-NF-κB when compared to LPS-treated control (Figure S2B). These results suggest that trehalose has the potential to inhibit LPS-induced inflammatory responses through TLR4-mediated NF-κB signaling.

### 3.4. Regulation of Macrophage Metabolic Phenotypes by Trehalose

**Figure 4:**
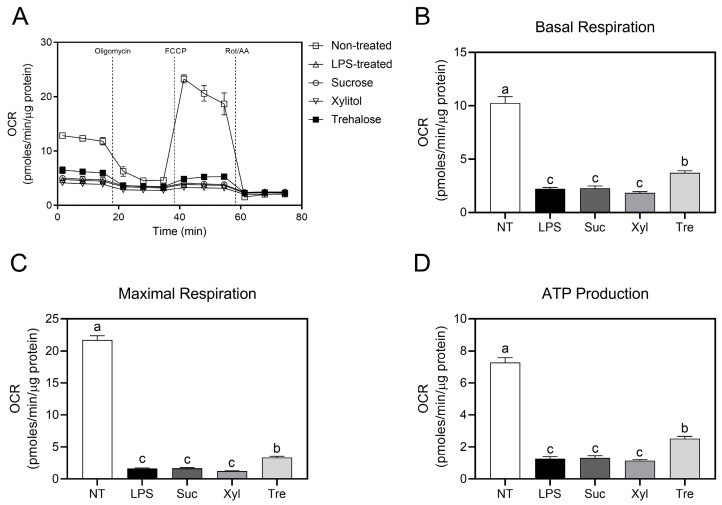
Mitochondrial respiratory phenotypes in LPS-treated macrophages. Following incubation with sucrose or sugar replacements and additional LPS stimulation, (**A**) real-time OCR of RAW 264.7 cells was calculated using a Seahorse extracellular flux analyzer by sequential treatment with three mitochondrial respiratory inhibitors. Based on OCR data, (**B**) the basal respiration, (**C**) maximal respiration, and (**D**) ATP production were calculated as described in the Materials and Methods, and normalized OCR is presented. Data are presented as mean ± SEM (*n* = 4). Different letters indicate significantly different values (*p* < 0.05) as determined by one-way ANOVA followed by Tukey’s posthoc test. NT, non-treated; Suc, sucrose; Xyl, xylitol; Tre, trehalose.

The real-time changes in OCR representing mitochondrial OXPHOS were measured using an extracellular flux analyzer to further study the effect of trehalose on energy metabolism in LPS-stimulated macrophages (Figure 4A). OCR profiles were analyzed for basal respiration, maximal respiration, and ATP production as described in Materials and Methods. LPS treatment induced a significant decrease in basal respiration (2.2 ± 0.14 pmoles/min/μg protein), maximal respiration (1.63 ± 0.08 pmoles/min/μg protein), and ATP production (1.26 ± 0.12 pmoles/min/μg protein) in macrophages compared to the untreated cells (basal respiration 10.26 ± 0.59 pmoles/min/μg protein, maximal respiration 21.72 ± 0.67 pmoles/min/μg protein, and ATP production 7.27 ± 0.32 pmoles/min/μg protein). Trehalose treatment exhibited significant upregulation in basal respiration (3.7 ± 0.18 pmoles/min/μg protein) as compared to sucrose (1.85 ± 0.23 pmoles/min/μg protein) and xylitol-treated cells (1.83 ± 0.11 pmoles/min/μg protein) (Figure 4B). Similarly, maximal respiration (3.34 ± 0.19 pmoles/min/μg protein) (Figure 4C) and ATP production (2.52 ± 0.35 pmoles/min/μg protein) (Figure 4D) of trehalose-treated cells exhibited significant increment as compared to sucrose (maximal respiration 1.66 ± 0.11 and ATP production 1.23 ± 0.06 pmoles/min/μg protein) and xylitol (maximal respiration 1.23 ± 0.06 and ATP production 1.14 ± 0.06 pmoles/min/μg protein).

**Figure 5:**
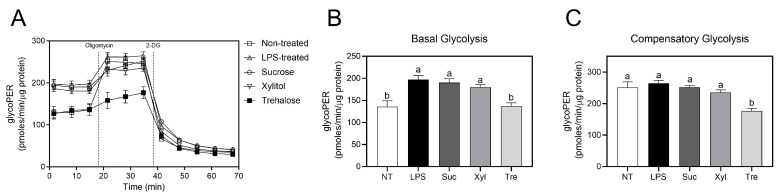
Glycolytic profiles of LPS-treated RAW 264.7 macrophages. (**A**) The glycolytic rate phenotype was measured using a Seahorse extracellular flux analyzer by consecutive injection with oligomycin and 2-deoxyglucose (2-DG). Continuous PER values normalized to total protein content are shown (pmoles/min/μg protein). (**B**) Basal glycolysis and (**C**) compensatory glycolysis were analyzed as explained in Materials and Methods. Data are presented as mean ± SEM (*n* = 4). Different letters indicate significantly different values (*p* < 0.05) as determined by one-way ANOVA followed by Tukey’s posthoc test. NT, non-treated; Suc, sucrose; Xyl, xylitol; Tre, trehalose.

Following the observation of altered oxygen consumption in macrophage mitochondria, the glycolytic functions of the cells were further investigated. Real-time changes in the glycolytic proton efflux rate (glycoPER), representing glycolytic phenotypes and further lactate fermentation, were calculated following sequential treatment with oligomycin and 2-DG (Figure 5A). Compared to non-treated cells (basal glycolysis 135.9 ± 13.03 pmoles/min/μg protein and compensatory glycolysis 251.1 ± 17.78 pmoles/min/μg protein), LPS treatment significantly enhanced basal glycolysis (197.3 ± 9.21 pmoles/min/μg protein), while compensatory glycolysis (264.6 ± 9.15 pmoles/min/μg protein) remained unaffected (Figure 5B,C). Trehalose significantly suppressed basal glycolysis (136.9 ± 7.84 pmoles/min/μg protein) compared to sucrose (190.3 ± 8.41 pmoles/min/μg protein) or LPS-treated control (Figure 5B). This homologous tendency was also observed in compensatory glycolysis (Figure 5C), in which trehalose significantly suppressed maximal glycolysis (176.7 ± 8.22 pmoles/min/μg protein) compared to sucrose (251.9 ± 6.26 pmoles/min/μg protein). In contrast, xylitol treatment did not show any significance to sucrose nor LPS-treated cells in both basal and compensatory glycolysis. Taken together, these results suggest that trehalose reciprocally regulates mitochondrial respiration and glycolysis in LPS-induced macrophages.

### 3.5. Suppression of Oxidative Stress by Trehalose Does Not Affect Mitochondrial Functioning

**Figure 6:**
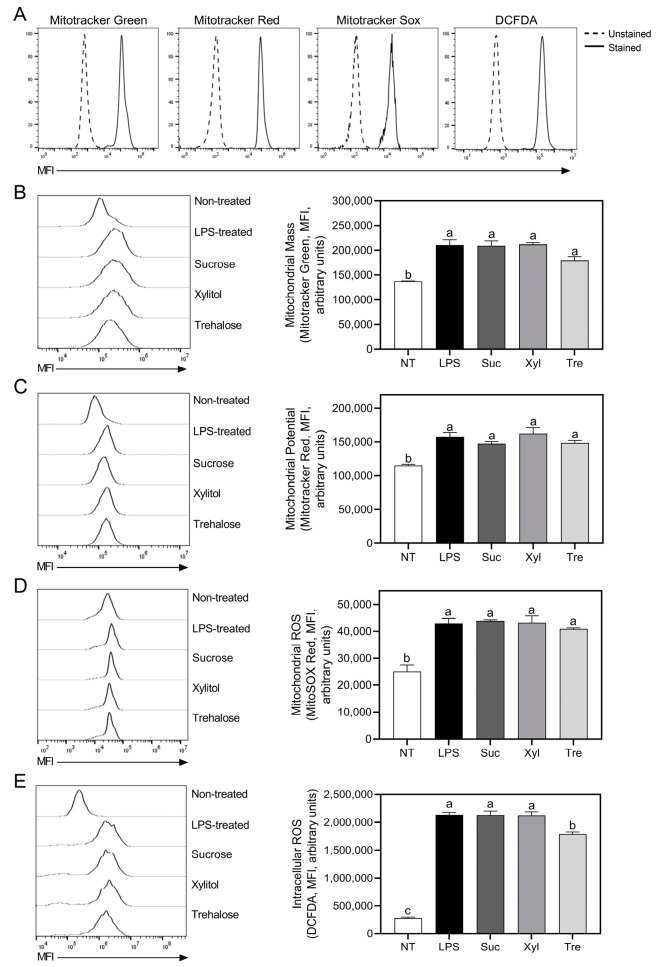
Analyses of mitochondrial functions and oxidative stresses in LPS-induced macrophages. (**A**) Mitotracker Green, Mitotracker Red, MitoSox, and DCFDA-stained cells were detected by flow cytometer (unstained cells-dashed-line, and stained cells-solid line), respectively. (**B**) Mitochondrial mass, (**C**) potential, and (**D**) ROS were measured through fluorescence intensity using a flow cytometer after staining RAW 264.7 cells with Mitotracker Green FM, Mitotracker CMXRos Red, and MitoSOX Red, respectively. (**E**) Intracellular ROS production was also analyzed by flow cytometry after staining the cells with DCFDA as described in Materials and Methods. Data are presented as mean ± SEM (*n* = 4). Different letters indicate significantly different values, and *p* < 0.05, as determined by one-way ANOVA followed by Tukey’s posthoc test. NT, non-treated; Suc, sucrose; Xyl, xylitol; Tre, trehalose.

To elucidate whether the alteration of mitochondrial function by trehalose affects macrophage energy metabolism, the mitochondrial mass, potential, and ROS were further measured (Figure 6A). Consistent with the observed increase in inflammatory mediator production, LPS treatment significantly elevated mitochondrial mass, potential, and intracellular ROS production compared to untreated cells (Figure 6B–E). As shown in Figure 6B, trehalose, as well as xylitol, did not affect the mitochondrial mass in macrophages as compared to sucrose. The mitochondrial potential was neither altered by trehalose nor xylitol (Figure 6C). A similar trend was observed for macrophage mitochondrial ROS production (Figure 6D), for which no significance was detected. Following the observation of the no effect of trehalose on mitochondrial oxidative stress, total ROS production was further assessed (Figure 6E). Interestingly, trehalose significantly down-regulated intracellular ROS production as compared to the sucrose control and xylitol treatment. Overall, these results suggest that the modulation of the immune response of macrophages by trehalose is not attributable to mitochondrial dysfunction but due to the reprogramming of energy metabolism.

## 4. Discussion

The current study sought to investigate the immune-regulating role of a commercial sugar replacement, trehalose. The results showed that trehalose exhibited suppressed LPS-induced inflammation in murine macrophages.

In comparison to widely used sucrose control, xylitol, a sugar alcohol, was also served as a negative control. Xylitol, a five-carbon sugar alcohol ((2*R*,3*R*,4*S*)-pentane-1,2,3,4,5-pentol), multiple hydroxyl (-OH) groups that aid in the scavenging of superoxide radicals. It was further reported to regulate metabolic syndromes, such as obesity, hyperlipidemia, and hyperglycemia [15,16,17]. In the current study, xylitol exhibited marginal effects in LPS-induced macrophages as assessed by the production of inflammatory mediators (Figure 2B,D), a well-established model for the quantification of inflammatory responses [18]. Specifically, PGE_2_, a pro-inflammatory lipid mediator produced from arachidonic acid by cyclooxygenase-2 (COX-2), was down-regulated by xylitol treatment as compared to sucrose control. However, the yield of NO, an arginine metabolite produced by inducible nitric oxide synthase (iNOS) [19,20], was not significant in xylitol-treated cells. The inhibition of PGE_2_ by xylitol may be relevant to their high radical scavenging ability as previously reported [21]. Despite moderate anti-inflammatory effects having been observed in xylitol-treated cells, there is a limitation to their application in nutraceuticals, as overconsumption may cause osmotic diarrhea [22,23,24].

In contrast to xylitol treatment, the anti-inflammatory effects of trehalose, a non-reducing disaccharide formed by two glucose units by α-1,1-glycosidic bond, were significantly observed. In detail, trehalose significantly suppressed the secretion of pro-inflammatory cytokines (IL-6 and TNF-α) (Figure 1) in macrophages. Furthermore, trehalose down-regulated the production of PGE_2_ and NO, as well as the transcription of their converting enzymes, COX-2 and iNOS, respectively (Figure 2). These results are in accordance with a previous study indicating that trehalose inhibits inflammatory cytokines, e.g., IL-1β and TNF-α, in LPS-induced mouse peritoneal macrophages [25]. In macrophage energy metabolism, trehalose significantly enhanced OXPHOS (Figure 4) while reciprocally down-regulating glycolysis (Figure 5). Trehalose also inhibited intracellular ROS production compared to sucrose (Figure 6), consistent with a previous study in which trehalose protected cellular stress by scavenging oxygen radicals [26]. Thus, trehalose has an active anti-inflammatory role by shifting energy metabolism from glycolysis to OXPHOS in LPS-induced macrophages without directly affecting the TLR4/NF-κB signaling pathway (Figure 3).

Taken together, the current study demonstrated that trehalose did not affect the aggravation of the LPS-induced macrophage inflammatory response. The anti-inflammatory effects of trehalose via reprogramming energy metabolism suggest that trehalose has the potential to serve as a tentative substitute for sucrose, a commonly consumed sugar. In particular, several studies have highlighted that trehalose is involved in the regulation of glycolysis by inhibiting hexokinase activity in yeast [27,28]. Consistent with these findings, the current study observed a comparable tendency in glycolysis, while confirming an elevation in oxidative phosphorylation. Consequently, further studies are required to elucidate the molecular mechanisms of macrophage phenotype switching by sugar replacements in inflammatory disease animal models.

## Figures and Tables

**Table 1:** Primer sequence for qRT-PCR.

Gene (NCBI ID)	Primer Sequence
*Eef2* (13629)	Forward, CGGGACACGGCTCTTAACAT Reverse, CTTCCTGGAGGCACTTACCC
*Il6* (16193)	Forward, CAAAGCCAGAGTCCTTCAGA Reverse, TTGGTCCTTAGCCACTCCTT
*Tnf-α* (21926)	Forward, AAATGGCCTCCCTCTCATCAG Reverse, GTCACTCGAATTTTGAGAAGATGATC
*Cox-2* (5912281)	Forward, TTCAAAAGAAGTGCTGGAAAAGGT Reverse, GATCATCTCTACCTGAGTGTCTTT
*inos* (18126)	Forward, CAGAGGACCCAGAGACAAGC Reverse, TGCTGAAACATTTCCTGTGC

## Data Availability

Data are contained within the article.

## Supplementary Materials

The following supporting information can be downloaded at: https://www.mdpi.com/article/10.3390/antiox12061166/s1, Figure S1: Effects of sucrose and sugar replacement on RAW 264.7 cell viability; Figure S2: Following RAW 264.7 cells were incubated with sucrose and sugar replacements and additional LPS treatment for 24 h.
