# Supplementary material for: Trehalose Inhibits Inflammatory Responses through Mitochondrial Reprogramming in RAW 264.7 Macrophages
Source: Antioxidants (Basel). 2023 May 28;12(6):1166. doi: 10.3390/antiox12061166 (PMC10295774; doi:10.3390/antiox12061166)
Supplement: Supplementary file 1 [file antioxidants-12-01166-s001.zip › antioxidants-2353886-supplementary.pdf]

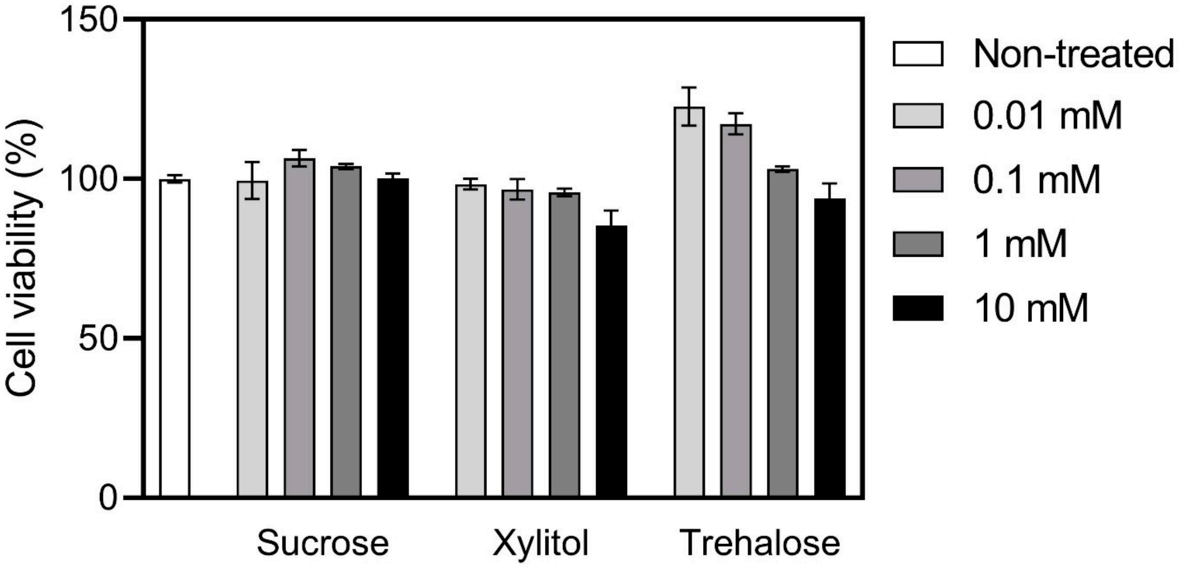

Figure S1. Effects of sucrose and sugar replacement on RAW 264.7 cell viability. The cell viability of RAW 264.7 macrophages following treatment of sucrose and sugar replacements was analysed by MTT assay. Cells were incubated with sucrose and sugar replacement at the designed concentration (0.1–100 mM), followed by incubation with MTT for additional 4 h. Results are expressed as mean ± SD (n = 4).

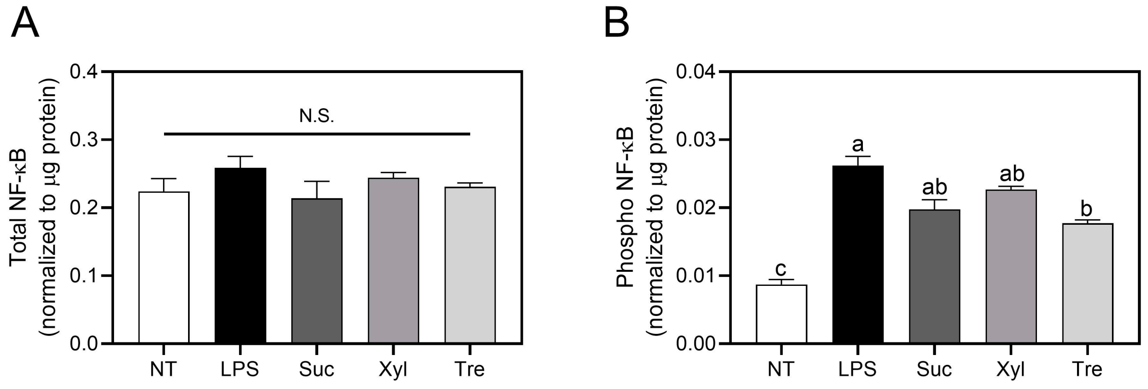

Figure S2. Following RAW 264.7 cells were incubated with sucrose and sugar replacements and additional LPS treatment for 24 h, the expression of (A) total NF-κB and (B) phospho- NF-κB was quantified by ELISA normalized to the amount of total proteins. Data are presented as mean SEM (n = 4). Different letters indicate significantly different values ($p < 0.05$) as determined by one-way ANOVA followed by Tukey's post-hoc test. NT, non-treated; Suc, sucrose; Xyl, xylitol; Tre, trehalose.
